# The effect of ovulation induction with clomiphene citrate on pregnancy outcomes in patients with subclinical hypothyroidism: A retrospective study

**DOI:** 10.12669/pjms.41.5.10535

**Published:** 2025-05

**Authors:** Esra Ince, Alev Atis, Aysu Akca, Lale Susan Karakis, Osman Ince

**Affiliations:** 1 Esra Ince, Gynecology and Obstetrics, Private Clinic, Antalya, Turkey; 2 Alev Atis, Department of Gynecology and Obstetrics, Sisli Etfal Training and Research Hospital, Istanbul, Turkey; 3 Aysu Akca, Department of Gynecology and Obstetrics, Memorial Sisli Hospital, Istanbul, Turkey; 4 Lale Susan Karakis, Gynecology and Obstetrics, Private Clinic, Istanbul, Turkey; 5 Osman Ince, Department of Gynecology and Obstetrics, Akdeniz University School of Medicine, Antalya, Turkey

**Keywords:** ART, Clomiphene Citrate, Hypothyroidism, Infertility, TSH

## Abstract

**Objectives:**

The literature indicates that there is no clear consensus on the effectiveness of assisted reproductive techniques (ART) in women with thyroid disorders. In this study, we aimed to compare the reproductive outcomes of patients taking clomiphene citrate (CC), focusing on those with normo-thyroid TSH concentrations (<2.5 µIU/ml) and those with subclinical hypothyroidism (2.5-5.0 µIU/ml).

**Methods:**

In this retrospective observational study, the medical records of 300 patients, who applied to the infertility department of Kanuni Sultan Süleyman Education and Research Hospital and administered CC between the years of January 2011 to December 2016 were examined. We analyzed the medical records of all women who took clomiphene citrate (CC) and had measured TSH levels between 2011 to 2016. Patients were divided into two groups as normo-thyroid and sub-clinic hypothyroidism. ART success was compared between the groups.

**Results:**

A total of 300 patients were included in the study, with 226 patients classified as normo-thyroid based on their TSH values at admission and 74 patients classified as having sub-clinic hypothyroidism. Among them, 123 normo-thyroid patients and 41 sub-clinic hypothyroid patients achieved pregnancy, with no significant difference found between the groups. Pregnancies were divided into groups as follows: singleton pregnancy, abortion, ectopic pregnancy, twin pregnancy and chemical pregnancy and then compared. The singleton pregnancy rate was 92.7% in normo-thyroid women and 85.4% in the sub-clinic hypothyroidism group with no statistically significant difference between the subgroups.

**Conclusion:**

Subclinical hypothyroidism does not alter pregnancy rates in women receiving CC for ovulation induction. In light of this study’s results, further research involving a greater number of patients from diverse ethnic backgrounds and various hospitals is needed to better understand the impact of subclinical hypothyroidism on reproductive outcomes in women taking CC.

## INTRODUCTION

Infertility is the inability to conceive for one year in women under the age of 35 and six months in women over the age of 35 despite unprotected sexual intercourse. In healthy couples, pregnancy occurs with a probability of 85-90% after one year of unprotected sexual intercourse. In other words, the frequency of infertility is 10-15% in couples of reproductive age.^1^ This rate reaches 25% in women in their 30s and increases further in women in their 40s.^2^ The most common causes of infertility are ovulatory dysfunction, tubal and peritoneal pathologies and male factors. Uterine pathology is relatively uncommon.

However, when no cause can be identified, the condition is referred to as unexplained infertility.^3^ Ovulation induction (OI) is one of the cornerstones of infertility treatment. Clomiphene citrate (CC) is one of the most commonly used agents for controlled ovarian stimulation (COH) in OI.^4^ CC belongs to the triphenyl ethylene group within the ‘Selective Estrogen Receptor Modulators’ (SERM). It can demonstrate both estrogenic and anti-estrogenic effects in different tissues. CC enhances gonadotrophin-releasing hormone (GnRH) release by pseudo-hypoestrogenic signals as a consequence of binding estrogen receptors at the level of the hypothalamus, correspondingly it enhances the release of gonadotrophins. Additionally, it is known that CC enhances the sensitivity of hypophysis to GnRh.^5^,^6^

Thyroid diseases such as subclinic hypothyroidism (SCH) are a very common disease group in Turkiye and globally. Women, who suffer from thyroid pathologies can have ovulatory disfunctions, menstrual irregularity, infertility and increased pregnancy complications.^7^ Hypothyroidism can enhance complications in pregnancy (neonatal mortality and morbidity, miscarriage, gestational hypertension, postpartum hemorrhage etc.).^1^,^8^-^10^ Although there is no clear consensus among endocrinologists and obstetricians about the TSH value during pregnancy, according to the American Thyroid Association, a TSH <2.5 µIU/ml is recommended.

No consensus on the success of assisted reproductive technologies (ART) in women with thyroid pathologies is available. Previous studies examining the interaction between SCH and ovarian insufficiency (OI) have demonstrated the effects of levothyroxine (LT4) treatment.^11^ However, the outcomes of women with asymptomatic SCH and euthyroid women have not been well investigated before initiating CC. This study aimed to compare pregnancy rates between patients who have SCH and *normothyroid* in patients in which OI with CC was applied.

## METHODS

In this retrospective observational study, the medical records of 300 patients, who applied to the infertility department of Kanuni Sultan Süleyman Education and Research Hospital and administered CC between the years of January 2011 to December 2016 were examined. Data were derived from medical records. Patients were divided into two groups during admission as normothyroid (TSH <2.5 mIU/L) (1st group) and SCH group (TSH ≥2.5 mIU/L and normal thyroid hormones) (2nd group).

### Ethical Approval:

This study was approved by the institutional review boards of Kanuni Sultan Süleyman Education and Research Hospital (Approval number: 2017-14-02, date: October 23, 2017) and the requirement for written informed consent was waived because of the retrospective design of the study. The study included women aged 18-39 years with ovulatory or unexplained infertility, who had regular sexual intercourse, a body mass index (BMI) <30 kg/m², no chronic co-morbidities, were not taking thyroid medications and had primary infertility. Exclusion criteria were infertility due to tubal factor or male factor and inadequate medical records. CC was administered with different dosages to patients from the 3rd-5th days of the cycle period and it was suggested that these doses be taken at the same time for five days one after the other.

Patients were called back in the mid-cycle phase according to their cycle period and the antral follicle was examined via transvaginal USG. Patients who had achieved sufficient follicular development (antral follicle size >18 mm) were advised to engage in intercourse every other day. Intercourse was prohibited if there was more than one dominant follicle present.^12^ Patients were called back to the hospital in the mid-luteal phase and serum progesterone values were reviewed. Ovulation was evaluated as positive in patients who had >3 µg/L and the same CC dosage was continued if pregnancy didn’t occur. In those patients in which progesterone levels were <3 µg/L, ovulation was evaluated as negative and the CC dosage was enhanced. Every patient who achieved human chorionic gonadotropin (B-HCG) positivity after taking CC for three or more cycles, regardless of the number of cycles, was included in the study. The primary endpoint of the study was achieving B-HCG positivity.

### Statistical Analysis:

The SPSS 25.0 (IBM Corporation, Armonk, New York, United States) program was used to analyze the variables. The conformity of the data to the normal distribution was evaluated using the Kolmogorov-Smirnov test. The Mann-Whitney U test was used together with the Monte Carlo results to compare two independent groups according to quantitative variables. In the comparison of categorical variables with each other, the Pearson Chi-Square and Fisher Freeman-Halton tests were tested with the Monte Carlo Simulation technique and column ratios were compared with each other and demonstrated according to p-value results with the Benjamini-Hochberg correction. Quantitative variables were expressed as Median (Minimum-Maximum) in the tables, while categorical variables are shown as n (%). The variables were analyzed at a 95% confidence level and a p-value of less than 0.05 was considered significant.

## RESULTS

A total of 300 patients were included in the study, comprising 226 patients (75.4%) with TSH values <2.5 uIU/L (1st group) and 74 patients (24.6%) with TSH values ≥2.5 uIU/L and normal thyroid hormones, indicating SCH (2nd group) at admission. The demographical and clinical features of patients are set out in Table-I. The mean CC dosage administered to patients was 61.6±27.2 mg in the 1st group, while in the 2nd group, the mean CC dosage administered was 66.2±28.7 mg. In the first group, the cause of infertility was anovulation in 92 patients (40.7%) with unexplained infertility present in 152 patients (59.3%).

**Table-I:** Demographic characteristics and pregnancy results of patients according to normothyroid group and SCH group.

	Total	TSH (2.5 µIU/ml)		p
		<2.5 µIU/ml	≥2.5 µIU/ml	
	(n:300)	(n:226)	(n:74)	
	Median (min-max)	Median (min-max)	Median (min-max)	
Age (year)	26 (19-37)	26 (19-37)	26 (19-36)	0.432^u^
	*n(%)*	*n(%)*	*n(%)*	
Age				0.743^c^
<30 years	238 (79.3)	178 (78.8)	60 (81.1)	
≥30 years	62 (20.7)	48 (21.2)	14 (18.9)	
Pregnancy Result (n-%)				0.149^f^
Ongoing pregnancy	149 (90.9)	114 (92.7)	35 (85.4)	
Abortus & Missed abortus	6 (3.7)	2 (1.6)	4 (9.8)	
Ectopic pregnancy	3 (1.8)	2 (1.6)	1 (2.4)	
Twin pregnancy	5 (3.0)	4 (3.3)	1 (2.4)	
Chemical pregnancy	1 (0.6)	1 (0.8)	0 (0.0)	
Infertility Reason (n-%)				0.177^c^
Unexplained	171 (57.0)	134 (59.3)	37 (50.0)	
Anovulation	129 (43.0)	92 (40.7)	37 (50.0)	
	*Median (min-max)*	*Median (min-max)*	*Median (min-max)*	
Cycle Number (n)	3 (1-6)	3 (1-6)	3 (1-6)	0.375^u^
Total Dose (CC-mg)	150 (50-900)	150 (50-900)	150 (50-600)	0.897^u^
	*n (%)*	*n (%)*	*n (%)*	
Cycle Number (n)				0.123^c^
<3	106 (35.3)	74 (32.7)	32 (43.2)	
≥3	194 (64.7)	152 (67.3)	42 (56.8)	
CC Dose (mg)				0.127^f^
25	1 (0.3)	1 (0.4)	0 (0.0)	
50	238 (79.3)	184 (81.4)	54 (73.0)	
100	43 (14.3)	27 (11.9)	16 (21.6)	
150	18 (6.0)	14 (6.2)	4 (5.4)	
Total Dose (mg)				0.783^c^
≤ 150 mg	184 (61.3)	140 (61.9)	44 (59.5)	
> 150 mg	116 (38.7)	86 (38.1)	30 (40.5)	

u Mann Whitney U test (Monte Carlo),

c Pearson Chi-Square test (Monte Carlo),

f Fisher-Freeman-Halton test (Monte Carlo),

or Odds Ratio (95% Confidence interval), Med.: Median, CC: Clomiphene Citrate, TSH: Thyroid stimulating hormone, mg: Miligram, min: Minimum, Max: Maximum, %: Percent, n: Number

In the second group, 37 patients (50%) had anovulation as the cause of infertility, while 37 patients (50%) had unexplained infertility. Patients in both groups exhibited similar distributions in terms of causes of infertility (p = 0.17). In dose comparison, CC doses were grouped as 25, 50, 100 and 150 mg as applied in the treatment protocols. Accordingly, no significant difference was found between the groups (p:0.127). When the cut-off value was separated as ≤150 mg and >150 mg, no statistically significant difference was observed (p:0.783). In addition, no statistically significant difference was found in the number of cycles performed (p:0.375). In the analysis of pregnancy outcomes, 123 patients (54.4%) in Group-I and 41 patients (55.4%) in Group-II became pregnant after treatment application. No statistically significant difference between rates of conception in the two groups was found either (p:0.149) (Table-I).

**Fig.1:**
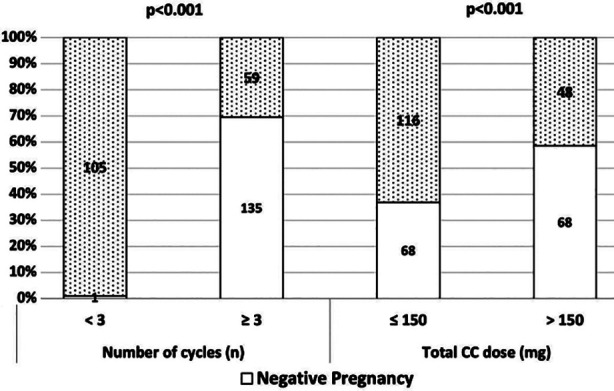
Pregnancy results according to CC dose and number of cycles.

When comparing the groups that achieved pregnancy and those that did not, no statistically significant difference was observed in terms of age, cause of infertility, TSH <2.5 mIU/L and ≥2.5 mIU/L (SCH) and CC dose administered (p>0.05). Statistically significant differences were observed between the number of cycles and total CC dose (p:0.001, 0.001) (Table-II, Fig.1). We believe that the reason for the increasing number of cycles and doses administered in the non-pregnant group was the increase in the number of trials as pregnancy did not occur.

**Table-II:** Demographic and treatment characteristics of patients according to pregnancy results.

	Pregnancy		p
	Negative	Positive	
	(n:136)	(n:164)	
	*Median (min-max)*	*Median (min-max)*	
Age (year)	26 (19-37)	25 (19-36)	0.300 ^u^
	*n (%)*	*n (%)*	
Age (year)			0.999 ^c^
<30	108 (79.4)	130 (79.3)	
≥30	28 (20.6)	34 (20.7)	
Infertility Reason			0.241 ^c^
Unexplained	83 (61.0)	88 (53.7)	
Anovulation	53 (39.0)	76 (46.3)	
TSH (µIU/ml)			0.894 ^c^
<2.5	103 (75.7)	123 (75.0)	
≥2.5	33 (24.3)	41 (25.0)	
	*Median (min-max)*	*Median (min-max)*	
TSH (µIU/ml)	1.615 (0.03-4.46)	1.795 (0.1-5.05)	0.428 ^u^
Cycle Number (n)	3 (2-6)	2 (1-6)	<0.001 ^u^
Total CC dose (mg)	175 (75-900)	100 (50-750)	<0.001^u^
	*n (%)*	*n (%)*	
Cycle number (n)			<0.001^c^
<3	1 (0.7)	105 (64.0)	240.3 (32.7-1762.7) ^or^
≥3	135 (99.3)	59 (36.0)	
CC dose (mg)			0.065 ^f^
25	1 (0.7)	0 (0.0)	
50	108 (79.4)	130 (79.3)	
100	15 (11.0)	28 (17.1)	
150	12 (8.8)	6 (3.7)	
Total CC Dose (mg)			<0.001^c^
≤150	68 (50.0)	116 (70.7)	2.4 (1.5-3.9) ^or^
>150	68 (50.0)	48 (29.3)	

u Mann Whitney U test (Monte Carlo),

c Pearson Chi-Square test (Monte Carlo),

f Fisher-Freeman-Halton test (Monte Carlo),

or Odds Ratio (95% Confidence interval), Med.: Median, CC: Clomiphene Citrate, TSH: Thyroid stimulating hormone, mg: Milligram, min: Minimum, Max: Maximum, %: Percent, n: Number

## DISCUSSION

SCH is characterized by elevated serum TSH levels, with serum thyroxine (T4) and serum triiodothyronine (T3) levels remaining within normal ranges. It is a common condition in adults. It is most common in thyroid hormone disorders. Although the association between thyroid pathologies and unfavourable pregnancy outcomes is clear, studies on the association between CC and OI are relatively limited. In this study, we compared the pregnancy outcomes of infertile women receiving OI with CC in the SCH and normothyroid groups.

In our study, SCH rates of 24.6% were likewise found in infertile women. In the study of Michalakis et al., 1231 women undergoing ART were evaluated.^13^ In this study, the SCH rate was found to be 23%. The study’s results revealed that low ovarian reversibility was linked to higher TSH levels during the preconceptional period. However, no association was found between TSH level and poor ART success and pregnancy outcome. We did not perform any analysis in terms of low ovarian reserve in our study. The most important reason for this is that at our clinic we started in-vitro fertilization (IVF) treatment instead of OI in women with low ovarian reserves.

Turgay et al. conducted an evaluation of the outcomes of IUI with euthyroid subfertile women in Türkiye.^14^ In the study, two groups were divided and compared based on TSH values: 0.5–2.49 mIU/L and 2.5–4.5 mIU/L, among women whose demographic data and cycle practices were similar. No statistically significant difference was found between the groups in terms of live birth rate (LBR), clinical pregnancy rates (CPR) and miscarriage rates. In a retrospective study conducted by Karmon et al. on 1477 patients, no significant difference was found between CPR and LBR in IUI results among euthyroid women, who had TSH values in the normal range (0.4-2.4 uIU/L) and those in the high-normal (2.5-4.9 uIU/L).^15^ Reh et al. established that a slight elevation in basal TSH levels (2.5-4.5 uIU/L) did not affect the CPR in their study involving 1055 patients undergoing IVF.^16^

Similarly, in our retrospective study and theirs, there was no significant difference found in CPR, delivery rates, or miscarriage rates between women with slightly elevated basal TSH levels compared to those with normal TSH levels (0.4-2.5 uIU/L). In the study of Aghahoseini et al., no statistically significant difference was found in terms of CPR in two groups divided based on TSH levels below and above 2.5 uIU/L in patients undergoing IVF.^17^ Moreover, there is ongoing discussion regarding the threshold of TSH levels that necessitate treatment for infertile women undergoing ART. The guideline of the Endocrine Society suggests that the upper limit of the TSH reference range must be 2.5 uIU/L in women, who were diagnosed before and have clinical hypothyroidism.^18^ However, it was proved that more frequent TSH checking did not result in an increase in CPR.^19^

Our study found no statistically significant difference in pregnancy rates (intrauterine pregnancy, ectopic pregnancy and twin pregnancy) between groups based on TSH level and when TSH level was compared to pregnancy occurrence. In a retrospective study that included 787 patients in Brazil, two groups were compared based on TSH levels below and above 2.5 mIU/L after intracytoplasmic sperm injection and a difference was not observed between live birth rates (LBR) or miscarriage rates.^20^ In a prospective cohort study, which Cai et al. undertook with 270 patients, the relationship between SCH and IVF results was examined. As a result, women receiving LT4 replacement therapy with basal TSH levels ranging from 0.2 to 2.5 uIU/L showed similar CPR, miscarriage rates and LBR compared to women with TSH levels ranging from 2.5 to 4.2 mIU/L.^19^

Our study is observational and the analysis of levothyroxine treatment has not been studied. In the future, we intend to conduct a study on the efficacy of levothyroxine treatment in our clinic. Baker et al. did not find any significant relation between TSH levels and spontaneous miscarriage in a retrospective study that was conducted with 195 patients, who practiced IVF. However, they observed lower gestational age and birth weight in women who became pregnant via IVF during cycle periods where TSH was above 2.5 mIU/L compared to cycles where TSH was below 2.5 mIU/L.^21^ Our study did not evaluate pregnancy outcomes, which is one of its limitations. The meta-analysis by Velkeiners et al. evaluating levothyroxine treatment in women with SCH undergoing ART is also of interest.^22^ In this meta-analysis, the results of 220 women were evaluated. They found that levothyroxine treatment had a positive effect on CPR, but there was insufficient data to evaluate the effect on preterm labour, hypertension, pre-eclampsia and detachment.

### Limitations:

The primary limitation of our study is that the data were collected retrospectively by reviewing patient files. Another limitation was the absence of data on late pregnancy outcomes such as live birth rates, as well as the lack of records for T3 and T4 hormone levels in our patients.

## CONCLUSION

No relationship between SCH and the occurrence of pregnancy was found in those patients taking CC. The effects of elevated TSH levels on reproductive outcomes in women are not known. Therefore, well-designed prospective studies that evaluate this topic are required.

### Abbreviations:

**ART:** Assisted reproductive techniques, **CC:** Clomiphene citrate, **OI:** Ovulation induction,

**COH:** Controlled ovarian stimulation, **SERM:** Selective Estrogen Receptor Modulators’,

**GnRH:** Gonadotrophin-releasing hormone, **IVF:** In-vitro fertilization, **BMI:** Body mass index, **LBR:** Live birth rate, **CPR:** Clinical pregnancy rates.

### Author Contributions:

**EI:** Original draft, formal analysis, writing.

**AAtis:** Data curation, project development.

**AAkca:** Study design and protocol, data curation

**LSK and OI:** Study design and protocol, data curation, writing, critical review & editing.

All authors have approved the final version of the manuscript.
